# Probabilistic polynomial dynamical systems for reverse engineering of gene regulatory networks

**DOI:** 10.1186/1687-4153-2011-1

**Published:** 2011-06-06

**Authors:** Elena S Dimitrova, Indranil Mitra, Abdul Salam Jarrah

**Affiliations:** 1 Department of Mathematical Sciences, Clemson University, Clemson, SC 29634-0975, USA; 2 Sealy Center of Molecular Medicine, University of Texas Medical Branch, Galveston, TX 77550, USA; 3 Virginia Bioinformatics Institute, Virginia Tech, Blacksburg, VA 24061-0477, USA; 4 Department of Mathematics and Statistics, American University of Sharjah, Sharjah, UAE

**Keywords:** Stochastic modeling, polynomial dynamical systems, reverse engineering, discrete modeling

## Abstract

Elucidating the structure and/or dynamics of gene regulatory networks from experimental data is a major goal of systems biology. Stochastic models have the potential to absorb noise, account for un-certainty, and help avoid data overfitting. Within the frame work of probabilistic polynomial dynamical systems, we present an algorithm for the reverse engineering of any gene regulatory network as a discrete, probabilistic polynomial dynamical system. The resulting stochastic model is assembled from all minimal models in the model space and the probability assignment is based on partitioning the model space according to the likeliness with which a minimal model explains the observed data. We used this method to identify stochastic models for two published synthetic network models. In both cases, the generated model retains the key features of the original model and compares favorably to the resulting models from other algorithms.

## Introduction

The enormous accumulation of experimental data on the activities of the living cell has triggered an increasing interest in uncovering the biological networks behind the observed data. This interest could be in identifying either the *static network*, which is usually a labeled directed graph describing how the different components of the network are wired together, or the *dynamic network*, which describes how the different components of the network influence each other. Identifying dynamic models for gene regulatory networks from transcriptome data is the topic of numerous published articles, and methods have been proposed within different computational frameworks, such as continuous models using differential equations [1,2], discrete models using Boolean networks [3], Petri nets [4-6], or Logical models [7,8], and statistical models using dynamic Baysein networks [9,10], among many other methods. For an up-to-date review of the state-of-the-art of the field, see, for example [11,12]. Most of these methods identify a particular model of the network which could be deterministic or stochastic. Due to the fact that the experimental data are typically noisy and of limited amount and that gene regulatory networks are believed to be stochastic, regardless of the used framework, stochastic models seem a natural choice [9,13,14]. Furthermore, discrete models where a gene could be in one of a finite number of states are more intuitive, phenomenological descriptions of gene regulatory networks and, at the same time, do not require much data to build. These models could actually be more suitable, especially for large networks [15].

The discrete modeling framework for gene regulatory networks that has received the most attention is *Boolean networks*, which was introduced by Kauffman [3]. They have been used successfully in modeling gene regulatory and signaling networks; see, for example [16-18]. Many reverse engineering methods have been developed to infer such networks, see, for example [19,20].

For the purpose of better handling noisy data and the uncertainty in model selection, Boolean networks were extended to *probabilistic Boolean networks *(PBN) in [13,21,22]. A PBN is a Boolean network where each node *i *may possibly have more than one Boolean transition function, say , where *t_i _*≥ 1, and, to decide the future state of *i*, a function  is chosen with probability *p_ij_*, where . To be precise, to each node i in a PBN, the set  of possible transition functions and their probabilities is assigned. Notice that if *t_i _*= 1 for all nodes in the network, then the PBN is just a Boolean network. As it is the case with Boolean networks, a PBN could be updated synchronously or asynchronously. However, throughout this article, we focus on synchronous PBN. Aspects of PBNs, and also asynchronous PBNs, have been studied in, for instance [23,24] and they have been applied to the modeling of gene regulatory networks in, for example, [25,26]. Furthermore, methods for inferring PBN have been developed in [27].

One disadvantage of Boolean models for gene regulatory networks is the limited number of states in which a gene can be. Indeed, although for a molecular biologist the state of a gene is usually discrete, it could be not only "expressed" and "not expressed" but also "over expressed," for example. There has thus been some consideration of more-than-binary discrete models in the Boolean network community. In the context of PBNs, generalizations of Boolean networks for ternary gene expression have been proposed in [28-31]. In addition, in [32] a ternary model has been considered as a preliminary stage for a Boolean one.

Other discrete multistate modeling frameworks have been developed too. Logical models [8] and *K*-bounded Petri nets [6,33] are two multistate modeling frameworks that have been used for modeling gene regulatory networks. A natural generalization of Boolean networks to multistate networks are the so-called polynomial dynamical systems (also known as algebraic models), which were introduced in [34]. In an algebraic model, the set of possible states of each node is a finite set, and once the mathematical structure of finite fields is imposed on that set, the transition function of each node is necessarily a polynomial. As this framework is rooted in computational algebra and algebraic geometry, results from these fields are used for the reverse engineering of dynamic and static biological networks [34-37], as well as for analyzing model dynamics [34,38], which usually is a challenge. Furthermore, in [39], it was shown that logical models and *K*-bounded Petri nets can be viewed as polynomial dynamical systems and algorithms for their translation into algebraic models were provided which facilitates the analysis of their dynamics.

In this article, we first introduce a stochastic generalization of polynomial dynamical systems, namely, *probabilistic polynomial dynamical systems*, which is also a generalization of the above-mentioned probabilistic Boolean networks to multistate models. Then, using this framework, we present a novel method for the reverse engineering of multistate gene regulatory networks from limited and noisy data. The novelty of our approach is two-fold. First, the stochastic model we construct is based on all minimal models in the model space and second, the probabilities assigned to the minimal models are based on an algebraic partition, called Gröbner fan, of the models space, which provides an algorithmic and algebraic method for the construction of such stochastic models.

In the next section, we present our method for the reverse engineering of gene regulatory networks as probabilistic polynomial dynamical systems. Then we demonstrate this method using the yeast cell cycle model in [17], as well as the synthetic network of the yeast cell cycle in [40].

## Methods

### Probabilistic polynomial dynamical systems

Laubenbacher and Stigler [34] proposed a modeling approach that describes a regulatory network on *n *genes as a deterministic polynomial dynamical system (PDS), i.e., a polynomial function (*f***_1_**, ..., *f_n_*): **K***^n ^*→ **K***^n^*, where **K **is a finite field. (*F *is just a Boolean network when **K **= {0, 1}.) Indeed, when **K **is a finite field, any function *F *: **K***^n ^*→ **K***^n ^*is a polynomial function, i.e., *F *can be described as (*f*_1_, ..., *f_n_*) where, for all *i*, *f*_*i *_: *k^n ^*→ *k *is a polynomial (see Appendix 1). This shows that PDSs are a suitable modeling framework naturally generalizing Boolean networks. We expand this framework to include stochastic models as follows.

**Figure 1:**
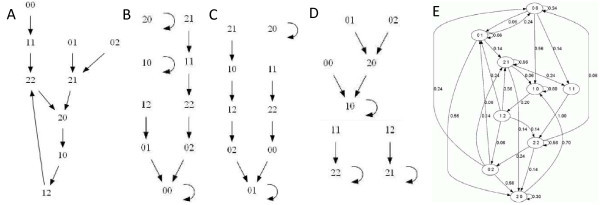
**The state spaces for the PPDS (1)**. **(A)**, **(B)**, **(C)**, and **(D) **deterministic state spaces induced by {*f*_11_, *f*_21_}, {*f*_11_, *f*_22_}, {*f*_12_, *f*_21_}, and {*f*_12_, *f*_22_}, respectively; **(E) **the stochastic state space induced by (**f_1_**, **f_2_**) with the probability of each transition labeled. All of these graphs are produced using the software Polynome [58].

A *probabilistic polynomial dynamical system *(PPDS) on *n *nodes is a polynomial function (**f_1_**, ..., **f_n_**) : **K***^n ^*→ **K***^n ^*where **K **is the set of possible sates of each node, and, for each node *i*,  is the set of functions that could be used to determine the future state of node *i *with probabilities . Given any state **x **= (*x*_1_, ..., *x_n _*) in state space **K***^n ^*of the system, the next state is determined as follows. For each node *i*, a local function *f_ij _*is selected from **f_i _**with probability *p_ij_*, and is used to compute the next state of node *i*, say *y_i_*. The set of all such transitions **x **→ **y **forms a directed graph, called the *state space *or *phase space*, on the vertex set **K***^n^*. For example, the PPDS , where(1)

and **F_3 _**= {0, 1, 2} is the finite field of three elements, is a PPDS whose state space (Figure 1E) has nine states. Notice that the state space of a PPDS is the union of the state spaces of all associated deterministic systems. In this example, as each node has two functions, there are four deterministic systems and their state spaces are in Figure 1A,B,C,D. For example, the state space of

is in Figure 1A.

### Reverse engineering PDSs

Laubenbacher and Stigler's reverse-engineering method [34] first constructs the set of all PDSs that fit the given discretized data, which we call here the model space, and then uses a minimality criterion to select one system from the model space. A unique feature of their method is that the model space is presented as an algebraic object. Their algorithm is summarized here as Algorithm 2.1.

Unless all state transitions of the system are specified, there will be more than one network that fits the given data set. Since this much information is hardly ever available in practice, any reverse-engineering method usually identifies one network model according to a pre-specified criterion, and different methods typically identify different models. In [34], first the set of all models is computed and then a particular one *f *= (*f*_1_, ... *f_n_*) is chosen that satisfies the following property: For each node *i*, the transition function *f_i _*is *minimal *in the sense that there is no non-zero polynomial *g *∈ *k *[*x*_1 _... *x_n_*] such that *f_i _*= *h *+ *g *and *g *is identically equal to zero on the given time points. This criterion for model selection is analogous to excluding the terms of *f_i _*that vanish on the data. The advantage of the polynomial modeling framework is that there is a well-developed algorithmic theory that provides mathematical tools for generating the model space as well as identifying the minimal models.

Algorithm 2.1 **Reverse engineering of PDSs**.

Input: A discrete time series of network states

Output: All minimal PDS's (*f*_1_, ..., *f_n_*) such that the *coordinate polynomials f_i _*∈ *k *[*x*_1_, ..., *x_n_*] satisfy *f*_*i *_(**s***_j_*) = *s*_*i,j*+1 _for all *i *= 1, ..., *n *and j = 1, ..., *m *- 1, and *f_i _*does not contain any term that vanish on the time series.

Step 1: Compute a PDS *f*_0 _: **K***^n ^***→ K***^n ^*that fits the data. There are several methods to do this, Lagrange interpolation being one of them.

Step 2: Compute the collection *I *of all polynomials that vanish on the data. Notice that if two polynomials *f_i_*, *g_i _*∈ *k *[*x*_1_, ..., *x_n _*] satisfy *f_i_*(**s***_j_*) = *s*_*i,j*+1 _= *g_i _*(**s***_j_*), then (*f_i _*- *g_i_*)(**s***_j_*) = 0 for all *j*. Therefore, in order to find all functions that fit the data, we need to find all functions that vanish on the given time points. Those functions form an algebraic object called the *ideal of points *and can be computed algorithmically.

Step 3: Reduce *f*_0 _= (*f*_1_, ..., *f_n_*) found in Step 1 modulo the ideal *I*. That is, write each *f_i _*as *f_i _*= *g *+ *h *with *h *∈ *I *and g being minimal in the sense that it cannot be further decomposed into *g *= *g*' + *h*' with *h*' ∈ *I*. In other words, *h *represents the part of *f_i _*that lies in *I *and is, therefore, identically equal to 0 on the given time series.

Algorithm 2.1 efficiently generates the set of all minimal PDS models that fit the data. However, identifying a single model may hardly be possible. There is a problem originating from Step 2 of Algorithm 2.1: finding all polynomials that vanish on a set of points. This is equivalent to computing the ideal of these points and computation of an ideal of points boils down to intersection of ideals. There is a well-known consequence of the Buchberger algorithm [41] for their computation. The output of the algorithm is a finite set of polynomials {*g*_1 _, ..., *g_s _*} ⊂ *k*[*x*_1_, ..., *x_n_*], called a *Gröbner basis *(for details see Appendix 2.1) that generates the ideal of vanishing on the data polynomials *I*:

The Gröbner basis, however, is not unique and its computation depends on the way the polynomial terms are ordered, called *monomial ordering *(Definition 2.1). The reason is that the remainder of polynomial division in polynomial rings in more than one variable is not unique and depends on the way the monomials are ordered. In contrast, this is not an issue in *k *[*x*] (a polynomial ring in one variable) where the monomials are ordered by degree: ⋯ ≻ *x*^*m*+1 ^≻ *x^m ^*≻ ⋯ *x*^2 ^≻ *x *≻ 1. However, whenever there is more than one variable, there is more than one choice for ordering the monomials (*e.g*., *x*^2 ^≻ *xy *and *xy *≻ *x*^2 ^are both possible) and thus the possibility of obtaining several different Gröbner bases. Consequently, the PDS model generated in Step 3 also depends on the choice of monomial ordering, as Example 3.1 illustrates.

Since different monomial orderings may give rise to different polynomial models, considering only one arbitrarily chosen monomial ordering is not sufficient. Therefore, a systematic method for studying the monomial orderings that affect the model selection is crucial for modeling approaches utilizing Gröbner bases. A naïve approach is to compute all possible Gröbner bases with respect to all monomial orderings. The number of monomial orderings, however, grows rapidly with the number of variables *n *and can be as large as *n*^2^*n*! [42] and hence considering all of them is computationally challenging. An alternative approach presented in [43] generates a collection of polynomial models from a fixed number of orderings (all graded reverse lexicographic) with random variable orderings and computes a consensus model using a game-theoretic method. While it is reasonable to try to avoid considering all monomial orderings, restricting oneself to variable orderings within a fixed monomial ordering will very likely miss a large number of PDS models that fit the data. Fortunately, the correspondence between Gröbner bases and monomial orderings is one-to-many. In [35], we presented a method which guarantees that no PDS model fitting the data is overlooked. Like [43], we avoided checking all possible monomial orderings but instead identified only those that produce *distinct *PDS models. The method is based on the combinatorial structure known as the *Gröbner fan *of a polynomial ideal which we discuss in more detail in Appendix 2.4. The Gröbner fan of an ideal *I *[44] is a polyhedral complex of cones with the property that every point encodes a monomial ordering. The cones are in bijective correspondence with the distinct Gröbner bases of *I*. (To be precise, the correspondence is to the marked reduced Gröbner bases of *I*). Therefore, it is sufficient to select exactly one monomial ordering per cone and, ignoring the rest of the orderings, still guarantee that all distinct models are generated. In addition, the relative number of monomial orderings under which a particular PDS model is generated provides an insight into the likelihood that the model is a good representation of the system; for details on this idea see Appendix 3. An excellent implementation of an algorithm for computing the Gröbner fan of an ideal is the software package *Gfan *[45].

### Algorithm for PPDS computation

We propose the following algorithm for the reverse engineering of gene regulatory networks as PPDS models from time series of discrete data. The resulting PPDS consists of all possible reduced PDS models that fit the data. The probability that we assign to each model is proportional to the relative volume of the Gröbner cone that produced that model. See Appendix 3 for assumptions and example.

Algorithm 3.1 **Reverse engineering of PPDSs**.

Input: A discrete time series of a gene regulatory network on *n *nodes *x*_1_, ..., *x_n_*: *S *= {(*s*_11 _, ..., *s*_*n*1_), ..., (*s*_1*m *_, ..., *s_nm_*)} ⊆ **K***^n^*, where **K **is a finite field.

Output: A probabilistic PDS model *F*, which is a list of all possible reduced local polynomials for each *x*_1_, ..., *x_n_*, together with their corresponding probabilities.

Step 1: Compute a particular PDS *F*_0 _: **K***^n ^*→ **K***^n ^*that fits *S*.

Step 2: Compute the ideal *I *of polynomials that vanish on *S*.

Step 3: Compute the Gröbner fan  of the ideal *I *and the relative sizes of its cones, *c*_1_, ..., *c_s _*(with *c*_1 _+ ⋯ + *c_s _*= 1).

Step 4: Select one (any) monomial ordering from each cone, ≺_1_, ..., ≺*_s_*. For each *i *= 1, ..., *s*, reduce *F*_0 _modulo *I *using a Gröbner basis computed with respect to ≺*_i_*. Let the reduced PDS's be *F*_1 _= {*f*_11_, *f*_12_, ..., *f*_1t_}, ..., *F_s _*= {*f*_*s*1_, *f*_*s*2_, ..., *f_st_*} and adding the cone sizes redefine them as *F_i _*= {(*f*_*i*1_, *c*_1_), (*f*_*i*2_, *c*_2_), ..., (*f_it_*, *c_t_*)}.

Step 5: Construct the list *F *= {{(*f*_11_, *c*_1_), (*f*_21 _, *c*_2_), ..., (*f*_*s*1_, *c_s_*)}, ..., {(*f*_1*t*_, *c*_1_), (*f*_2*t*_, *c*_2_), ..., (*f_st_*, *c_s_*)}}. For a fixed *i*, if *f_ji _*= *f_ki _*for some *j *and *k*, then "merge" the two local polynomials by adding their corresponding probabilities: (*f_ji_*, *c_j _*+ *c_k_*).

Algorithm 3.1 guarantees that all distinct minimal PDS models will be generated. However, this comes at the expense of having to compute the entire Gröbner fan of the ideal of points. For small networks the computation of the fan is feasible but as the number of network nodes increases, the complexity of the Gröbner fan computation becomes prohibitive [46]. As mentioned earlier, the correspondence between PDS models and Gröbner bases is one-to-many. Therefore, computing the entire Gröbner fan of the ideal of vanishing polynomials is excessive and instead a finite subset of points from the fan should be sufficient. This finite subset needs to be carefully selected if we want it to reflect the structure of the entire Gröbner fan. Since we want to rank the dependencies according to their strength, the number of points (weight vectors) we select from a Gröbner cone should correspond to the relative size of this cone with respect to the other cones. That is, we want to sample from the Gröbner fan uniformly, so that the relative frequency with which we select term orders from the fan is approximately equal to the relative sizes of its cones. We do this through random sampling of the Gröbner fan of the ideal of points as in [47]. If the number of points is sufficiently large, their distribution approximately reflects the relative size of the Gröbner cones. The number of points is determined using a *t *test for proportion. Consequently, steps 3 and 4 of Algorithm 3.1 have to be modified in such a way that direct computation of the Gröbner fan is avoided.

Step **3**': Select vectors **w**_1_, ..., **w***_s _*of length *n*, with *s *large, in such a way that every (nonnegative integer) vector in the Gröbner fan of *I *has equal probability of being chosen.

Step **4**': For each *i *= 1, ..., *s*, use **w***_i _*to define a monomial ordering ≺*_i _*and reduce *F*_0 _modulo *I *using a Gröbner basis computed with respect to ≺*_i_*.

## Examples and results

### Reverse engineering of the yeast cell cycle

We applied the PPDS method to the reverse engineering of the gene regulatory network of the cell cycle in *Saccharomyces cerevisiae *starting from a data set generated from the well-known discrete model suggested by Li et al. [17]. The cell cycle is the process of cell growth and division and consists of four phases. The cell cycle in *S. cerevisiae *has been extensively studied and about 800 genes are known to participate in the process. It is believed, however, that the number of key regulators is much smaller and, based on an extensive literature review [17] constructed a Boolean network on 11 distinct nodes: Cln3, MBF, SBF, Cln1, 2, Cdh1, Swi5, Cdc20 and Cdc14, Clb5, 6, Sic1, Clb1, 2, Mcm1/SFF. For the network dynamics, a threshold function is assigned to each node in the network according to (2), where *a_ij _*represents the weight of effect of node *j *on node *i*.(2)

This model captures the known features of the cell cycle dynamics. Furthermore, the trajectory of the known cell cycle sequence is stable and attracting, as its size is 1764 out of the total of 2048 states. The remaining states are distributed into 6 very small trajectories. Each of these trajectories converges to a steady state as well.

We used as input to our Algorithm 3.1 54 input-output transitions, four of which are steady states (see Table 1). Our reverse engineering algorithm generated the PPDS (6). The state space of this system consists of 14 connected components, where each component ends in a steady state. The built-in four steady states belong to components of sizes very close to those of the original system. In addition, the other three steady states in the original system were also recovered. These results are summarized in Table 2. The seven steady states of our model, which are not in the original system, with one exception belong to very small components (less than 30 points).

**Table 1:** The 54 state transitions used for generating the PPDS model (6), each represented by a pair of input-output states

Cln3	MBF	SBF	Cln1, 2	Cdh1	Swi5	Cdc20 and Cdc 14	Clb5, 6	Sic1	Clb1, 2	Mcm1/SFF	Cln3	MBF	SBF	Cln1, 2	Cdh1	Swi5	Cdc20 and Cdc14	Clb5, 6	Sic1	Clb1, 2	Mcm1/SFF
**0**	**0**	**0**	**0**	**0**	**0**	**0**	**0**	**0**	**0**	**0**	0	0	0	0	0	1	1	0	0	0	0
**0**	**0**	**0**	**0**	**0**	**0**	**0**	**0**	**0**	**0**	**0**	0	0	0	0	1	1	0	0	1	0	0
**0**	**0**	**0**	**0**	**1**	**0**	**0**	**0**	**0**	**0**	**0**	0	0	0	0	0	1	0	0	0	1	0
**0**	**0**	**0**	**0**	**1**	**0**	**0**	**0**	**0**	**0**	**0**	0	0	0	0	0	0	1	0	0	1	1
**0**	**1**	**0**	**0**	**0**	**0**	**0**	**0**	**1**	**0**	**0**	0	0	0	0	0	0	0	0	1	0	1
**0**	**1**	**0**	**0**	**0**	**0**	**0**	**0**	**1**	**0**	**0**	0	0	0	0	0	1	1	0	1	0	0
**0**	**0**	**1**	**1**	**0**	**0**	**0**	**0**	**0**	**0**	**0**	1	1	0	0	0	0	0	0	0	1	0
**0**	**0**	**1**	**1**	**0**	**0**	**0**	**0**	**0**	**0**	**0**	0	1	0	0	0	0	1	1	0	1	1
0	0	1	0	0	0	0	0	0	0	0	0	0	0	0	0	1	0	1	0	0	0
0	0	1	1	0	0	0	0	0	0	0	0	0	0	0	0	0	0	1	0	1	1
0	0	0	1	0	0	0	0	0	0	0	0	0	0	0	0	1	0	0	1	0	0
0	0	0	0	0	0	0	0	0	0	0	0	0	0	0	0	0	0	0	1	0	0
0	0	0	0	0	1	0	0	0	0	0	0	0	0	0	0	1	0	0	0	0	1
0	0	0	0	0	0	0	0	1	0	0	0	0	0	0	0	1	1	0	1	1	0
0	0	0	0	0	0	1	0	0	0	0	0	0	0	0	0	0	1	1	0	0	0
0	0	0	0	1	1	0	0	1	0	0	0	0	0	0	0	1	0	0	0	0	1
0	0	0	0	0	0	0	1	0	0	0	0	0	0	0	0	0	1	0	1	0	0
0	0	0	0	0	0	0	1	0	1	1	0	0	0	0	1	1	0	0	1	0	0
0	0	0	0	0	0	0	0	0	1	0	0	0	0	0	0	0	1	0	0	1	0
0	0	0	0	0	0	1	0	0	1	1	0	0	0	0	0	0	1	0	0	0	1
0	0	0	0	0	0	0	0	0	0	1	0	0	0	0	0	0	1	0	0	0	1
0	0	0	0	0	1	1	0	0	1	0	0	0	0	0	1	1	1	0	1	0	0
1	1	0	0	0	0	0	0	0	0	0	0	0	0	0	0	0	0	1	1	0	0
0	1	1	0	0	0	0	1	0	0	0	0	0	0	0	0	0	0	0	0	0	1
1	0	1	0	0	0	0	0	0	0	0	0	0	0	0	0	0	0	1	0	1	0
0	1	1	1	0	0	0	0	0	0	0	0	0	0	0	0	0	1	1	0	1	1
0	1	0	0	0	0	1	0	0	0	0	0	0	0	0	0	0	0	1	0	0	1
0	1	0	0	1	1	0	0	1	0	0	0	0	0	0	0	1	1	1	0	1	1
0	1	0	0	0	0	0	1	0	0	0	0	0	0	0	0	0	0	0	1	1	0
0	1	0	0	0	0	0	1	0	1	1	0	0	0	0	0	0	1	0	0	0	1
0	1	0	0	0	0	0	0	0	1	0	0	0	0	0	0	0	0	0	0	1	1
0	0	0	0	0	0	1	1	0	1	1	0	0	0	0	0	0	1	0	0	1	1
0	0	1	0	0	0	0	0	0	1	0	1	0	1	0	0	0	0	0	0	1	0
0	0	0	1	0	0	1	0	0	1	1	0	0	1	1	0	0	1	0	0	1	1
0	0	0	1	1	0	0	0	0	0	0	0	0	0	1	1	0	1	0	0	0	0
0	0	0	0	0	0	0	0	0	0	0	0	0	0	0	1	1	0	0	0	0	0
0	0	0	1	0	1	0	0	0	0	0	0	0	0	0	1	0	1	1	0	0	0
0	0	0	0	0	0	0	0	0	0	0	0	0	0	0	1	1	0	0	0	0	1
0	0	0	1	0	0	1	0	0	0	0	0	0	0	1	1	0	0	0	0	1	0
0	0	0	0	0	1	0	0	0	0	0	0	0	0	0	0	0	1	0	0	0	1
0	0	0	1	0	0	0	1	0	0	0	0	0	0	1	0	0	1	1	0	0	0
0	0	0	0	0	0	0	1	0	1	1	0	0	0	0	0	1	0	0	0	0	1
0	0	0	1	0	0	0	0	1	0	0	0	0	0	1	0	0	1	0	0	1	0
0	0	0	0	0	0	0	0	0	0	0	0	0	0	0	0	0	1	0	0	0	1
0	0	0	1	0	0	0	0	0	1	0	0	0	0	1	0	0	0	1	0	1	0
0	0	0	0	0	0	1	0	0	1	1	0	0	0	0	0	0	1	1	0	1	1
0	0	0	0	1	0	1	0	0	0	0	0	0	0	0	1	0	1	1	0	0	0
0	0	0	0	1	1	0	0	1	0	0	0	0	0	0	1	1	0	0	0	0	1
0	0	0	0	1	0	0	1	0	0	0	0	0	0	0	1	0	1	0	0	1	0
0	0	0	0	0	0	0	1	0	0	1	0	0	0	0	1	0	1	0	0	0	1
0	0	0	0	1	0	0	0	0	1	0	0	0	0	0	1	0	0	1	0	1	0
0	0	0	0	0	0	1	0	0	0	1	0	0	0	0	0	0	1	1	0	1	1
0	0	0	0	1	0	0	0	0	0	1	0	0	0	0	0	0	1	1	0	1	0
0	0	0	0	1	1	1	0	0	0	0	0	0	0	0	0	0	1	0	0	1	1

The four fixed points are listed first in bold. The transitions are generated from the 11 variable model of Li et al. [17].

**Table 2:** Comparison of the steady states of model (2) and those of the probabilistic PDS (6) built via our reverse engineering method using the data set in Table 1 generated from model (2)

Fixed point	Is it input?	Original system component size	Reverse engineered component size
1	No	1, 764	1, 015
2	Yes	151	70
3	No	109	9
4	No	9	9
5	Yes	7	7
6	Yes	9	5
7	Yes	1	1

Further, we assessed the quality of the dependency graph of the inferred model using three standard network measures: *positive predictive value*, PPV = TP/(TP + FP) = 0.83, *specificity *, Sp = TN/(TN + FP) = 0.94, and *sensitivity*, Se = TP/(TP + FN) = 0.69, where TP and TN are the numbers of true positive and negative interactions, respectively, and FP and FN are the numbers of false positive and false negative interactions, respectively, weighted by the corresponding probabilities given after every polynomial in (6). The high values of the three measures indicate that the proposed method is not only capable of capturing the dynamic behavior of the system but also its static wiring network.

### Comparison to other methods

**Figure 2:**
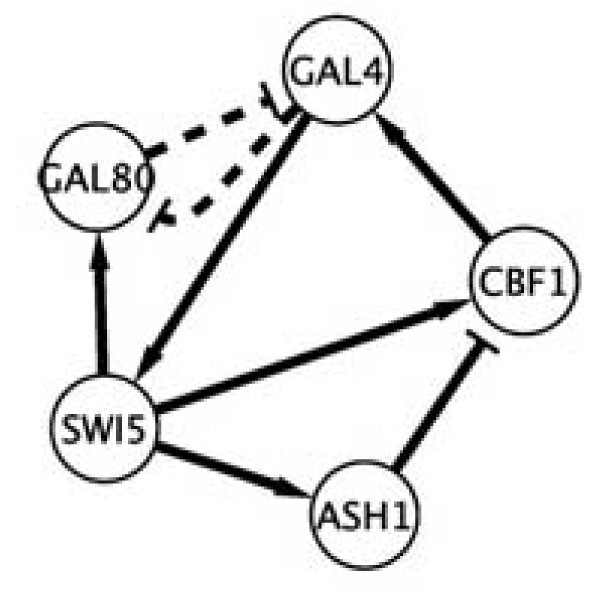
**The five gene synthetic networks in *S. cerevisiae *built by Cantone et al. **[40].

We also performed a comparison of our algorithm to several other reverse engineering methods. In [40], Cantone et al. built in *S. cerevisiae *a synthetic network for in vivo "benchmarking" of reverse-engineering and modeling approaches. The network in Figure 2 is composed of five genes (CBF1, GAL4, SWI5, GAL80, and ASH1) that regulate each other through a variety of regulatory interactions. The mathematical model of the network is based on nonlinear differential equations obtained from standard mass-balance kinetic laws. Time series and steady-state expression data were measured after multiple perturbations. In particular, they performed perturbation experiments by shifting cells from glucose to galactose ("switch-on" experiments) and from galactose to glucose ("switch-off" experiments). The synthetic network was then used to assess the ability of experimental and computational approaches to infer regulatory interactions from gene expression data. Four published algorithms were selected as representatives of reverse-engineering approaches: BANJO (Bayesian networks) [48], NIR and TSNI (ordinary differential equations) [49,50], and ARACNE (information theory) [51]. These methods were assessed based on their positive predictive value (PPV) and sensitivity (Se). In order to test the significance of the algorithms, the "random" performance was computed, which refers to the expected performance of an algorithm that randomly assigns edges between a pair of genes. For example, for a fully connected network, the random algorithm would have a 100% accuracy (PPV = 1) for all the levels of sensitivity (as any pair of genes is connected in the real network). For the net-work in Figure 2, the expected PPV for a random guess of directed interactions among genes is PPV = 0.40, so any value higher than 0.4 will be significant. (In the case of undirected interactions, the random guess has PPV = 0.70.)

Using the same data sets, which we discretized into three states applying the algorithm in [52], our method (PPDS) performed well when compared to the best method (the ordinary differential equations approach TSNI) according to [40]. A summary is given in Table 3. Notice that although the PPV value of PPDS on the switch-on data is lower than that of TSNI, it is still well above 0.40 and thus it is better than random.

**Table 3:** PPV, positive predictive value and Se, sensitivity of the reverse-engineering approaches NIR, TSNI, BANJO, ARACNE, and PPDS when applied to data generated from the synthetic network in [40]

	Switch-on		Swith-off	
	**PPV**	**Se**	**PPV**	**Se**

NIR and TSNI	0.80	0.50	0.6	0.38
BANJO	*	*	0.6	0.38
ARACNE	*	*	*	*
PPDS	0.57	0.50	0.75	0.38

The symbol * stands for "worse than random."

## Conclusion

Gene regulatory networks are structured as inter-connected entities and their complex nature is inherently stochastic. The framework of stochastic dynamical systems is natural for modeling and analyzing such networks. We focused on PPDSs due to their applicability to limited and possibly noisy data. Within this modeling framework, we developed a systematic method based on combinatorial topology, algebraic geometry, and statistics for the reverse engineering of the dynamics, as well as the gene dependencies, in biochemical regulatory networks from experimental data. The algorithm can handle large regulatory networks and hence is applicable to many networks of interest. The constructed models are comprised of minimal polynomials according to the definition in [34]. We plan to explore the use of other types of biologically relevant functions, such as nested canalyzing functions [53]. An algorithm for the inference of deterministic nested Boolean canalyzing networks has recently been presented (F Hinkelmann, A Jarrah: Inferring biologically relevant models: nested canalyzing functions, submitted). Combining this with our algorithm here will provide a systematic method for the reverse engineering of gene regulatory networks as probabilistic Boolean nested canalyzing networks.

## Abbreviations

PDS: polynomial dynamical system; PPV: predictive value; PBN: probabilistic Boolean networks; PPDS: probabilistic polynomial dynamical system; Se: sensitivity.

## Competing interests

The authors declare that they have no competing interests.

## Appendices

### 1 Polynomial dynamical systems

**Definition 1.1 **Let *X *be a finite set. A **finite dynamical system **of dimension *n *is a function *F *= (*f*_1_, ..., *f_n_*) : *X^n ^*→ *X^n ^*with *f_i _*: *X^n ^*→ *X*.

By requiring that the cardinality of the set *X *be a power of a prime number, one can impose on *X *the structure of a *finite field*. This structure determines the only type of functions *f_i _*that need to be considered. The following theorem from [54] characterizes functions over finite fields.

**Theorem 1.1 ***Let k be a finite field. Then every function f : k^n ^→ k is a polynomial of degree at most n*.

Therefore, over a finite field, polynomials are the appropriate modeling framework rather than a constraining assumption.

**Definition 1.2 **If the set *X *for a finite field, then any function *F *: *X *→ *X *is called a **polynomial dynamical system **(PDS).

**Definition 1.3 **A **probabilistic polynomial dynamical system **(PPDS) on *n *nodes (**f_1_**, ..., **f_n_**) : **K***^n ^***→ K***^n ^*with parallel update order consists of *n *sets of local functions and their associated probabilities such that  is the set of local functions that determine the dynamics of node *i *and . In order to determine each transition in the state space of the system, (*x*_1_, ..., *x_n_*) **→ **(*y*_1_, ..., *y_n_*), for each node *i *a local function *f_ij _*is selected from **f_i _**with probability *p_ij_*.

As an example, see (1).

### 2 Concepts from commutative algebra and algebraic geometry [55]

#### 2.1 Gröbner bases

A polynomial in *k*[*x*_1_, ..., *x_n_*] is a linear combination of monomials of the form  over *k*, where α is the *n*-tuple exponent . For many purposes, such as polynomial division, it is necessary to arrange the terms in a polynomial unambiguously in some order. Unlike polynomials in one variable, there are more than one way of ordering the terms (monomials) of multivariate polynomials. Any ordering of the monomials must be a *total *ordering, *i.e*., for every pair of monomials *x^α ^*and *x^β^*, exactly one of the following must be true: *x^α ^*≺ *x^β^*, *x^α ^*= *x^β^*, *x^α ^*≻ *x^β^*. Taking into account the properties of the polynomial sum and product operations, the following definition emerges.

**Definition 2.1 **A **monomial ordering **on *k*[*x*_1_, ..., *x_n_*] is any relation ≻ on  satisfying:

1. ≻ is a total ordering on .

2. If *α *≻ *β *and , then *α *+ *γ *≻ *β *+ *γ*.

3. ≻ is a well-ordering on , *i.e*., every nonempty subset of  has a smallest element under ≻.

A monomial ordering can also be defined by a weight vector *ω *= (*ω***_1_**, ..., *ω_n_*) in . We require that *ω *have nonnegative coordinates in order for 1 to always be the smallest monomial. Fix a monomial ordering ≻*_σ_*, such as ≻*_lex_*. Then, for , define α ≻ *_ω,σ _β *if and only if *ω *· α ≻ *ω *· *β*, or *ω *· *α *= *ω *· *β *and α ≻*_σ _β*.

**Ideal membership problem **Another problem with multivariate polynomial division is that when dividing a given polynomial into more than one polynomials, the outcome may depend on the order in which the division is carried out. Let *f*, *g*_1_, ..., *g_m _*∈ *k *[*x*_1_, ..., *x_n_*] be polynomials in the variables *x*_1_, ..., *x_n_*. The so-called *ideal membership problem *is to determine whether there are polynomials *h*_1_, ..., *h_m _*∈ *k*[*x*_1_, ..., *x_n _*] such that . To state this in the language of abstract algebra, we define *I *= 〈*g*_1_, ..., *g_m_*〉 := {∑*h_i_g_i _*| *h*_1_, ..., *h_m _*∈ *k*[*x*_1_, ..., *x_n_*]} . The polynomials in I form a so-called *ideal *in *k*[*x*_1_, ..., *x_n_*], since *I *is closed under addition and multiplication by any polynomial in *k*[*x*_1_, ..., *x_n_*], and *I *is generated by the set {*g*_1_, ..., *g_m_*}. The ideal membership problem asks if *f *is an element of *I *. In general, even under a fixed monomial ordering, the order in which *f *is divided by the generating polynomials *f_i _*affects the remainder . Therefore,  does not imply *f *∉ *I*. Moreover, the generating set {*f*_1_, ..., *f_m_*} of the ideal *I *is not unique but a special generating set  can be selected so that the remainder of polynomial division of *f *by the polynomials in  performed in any order is zero if and only if *f *lies in *I*: . A generating set with this property is called a *Gröbner basis *and its precise definition will be given in Definition 2.3. Here we point out that Gröbner bases provide an algorithmic solution to the ideal membership problem and the Buchberger algorithm [41] is designed to compute a Gröbner basis for any ideal other than {0} and a fixed monomial ordering.

#### 2.2 Monomial Ideals

Gröbner bases are a key concept in computational algebra. Their theory reduces questions about systems of polynomial equations to the combinatorial study of *monomial ideals*.

**Definition 2.2 **An ideal *I *⊂ *k*[*x*_1_, ..., *x_n_*] is a **monomial ideal **if *I *is generated by monomials, i.e., there is a subset  such that *I *= 〈*x^α ^*| *α *∈ *A*〉, *i.e*., consists of all polynomials which are finite sums of the form , where *h_α _*∈ *k*[*x*_1_, ..., *x_n_*].

A special kind of monomial ideal is the *initial ideal *of an ideal *I *≠ {0} for a fixed monomial ordering. It is the ideal generated by the set of initial monomials (under the specified ordering) of the polynomials of *I*: *in *(*I*) = 〈*in*(*f*) | *f*∈ *I*〉 . The monomials which do not lie in *in*(*I*) are called *standard monomials*.

**Definition 2.3 **Fix a monomial ordering. A finite subset  of an ideal *I *is a **Gröbner basis **if .

A Gröbner basis for an ideal may not be unique. If we also require that for any two distinct elements , no term of g' is divisible by *in*(*g*), such a Gröbner basis is called *reduced *and is unique for an ideal and a monomial ordering, provided the coefficient of *in*(*g*) in g is 1 for each 

#### 2.3 Ideals of points

Given a set of points, it is often necessary to find all the polynomials that vanish on it. Such a set of polynomials forms an ideal called the *ideal of points *defined as follows.

**Definition 2.4 **Let *V *= {*p*_1_, ..., *p_m_*}, where *p_i _*= (*a*_*i*1_, ..., *a_in_*) ∈ *k^n^*. Then we set

It can be shown that  is an ideal of *k*[*x*_1_, ..., *x_n_*]. It is called the *ideal of points *in *V *.

#### 2.4 The Gröbner fan of an ideal

A combinatorial structure that contains information about the initial ideals of an ideal is the *Gröbner fan *of an ideal. It is a polyhedral complex of cones, each corresponding to an initial ideal, which, as follows from Definition 2.3, is in a one-to-one correspondence with the *marked *reduced Gröbner bases (the initial term of each generating polynomial being distinguished) of the ideal. A brief introduction to the the Gröbner fan folllows. For details see, for example [44].

A polynomial ideal has only a finite number of different reduced Gröbner bases. Informally, the reason is that most of the monomial orderings only differ in high degree and the Buchberger algorithm for Gröbner basis computation does not "see" the difference among them. However, they may vary greatly in number of polynomials and "shape". In order to classify them, we first present a convenient way to define monomial orderings using matrices [56]. Again, we think of a polynomial in *k*[*x*_1_, ..., *x_n_*] as a linear combination of monomials of the form  over *k*, where *α *is the *n*-tuple exponent .

**Definition 2.5 **Let *ω *= (*ω*_1_, ..., *ω_n_*) be a vector with real coefficients. We can define an ordering ≻*_ω _*the elements of  by *α *≻*_ω _β *if and only if *α *· *ω *>*β *· *ω*, componentwise.

**Definition 2.6 **Let  be a marked reduced Gröbner basis for an ideal *I*. Write each polynomial of the basis as  where  is the initial term in *g_i_*. The **cone **of  is 

The collection of all the cones for a given ideal is the **Gröbner fan **of that ideal. The cones are in bijection with the marked reduced Gröbner bases of the ideal. Since reducing a polynomial modulo an ideal *I*, as the reverse engineering algorithm requires in Step 3, can have at most as many outputs as the number of marked reduced Gröbner bases, it follows that the Gröbner fan contains information about *all *Gröbner bases (and thus all monomial orderings) that need to be considered in the process of model selection. There are algorithms based on the Gröbner fan that enumerate all marked reduced Gröbner bases of a polynomial ideal [45].

### 3 Reverse engineering of PPDSs

Suppose we have time series data from a gene regulatory network on *n *genes represented by variables *x*_1_, ..., *x_n_*. Let *f *= (*f*_1_, ... *f_n_*) be any polynomial system that fits the data, generated using, for instance, Lagrange interpolation, and suppose that variable *x_i _*appears in at least one monomial (with a nonzero coefficient) of polynomial *f_j_*. Then it follows that variable *x_i _*has effect on variable *x_j _*whose behavior is determined by *f_j_*. The directed graph on {*x*_1_, ..., *x_n_*} representing these dependencies is called the *dependency graph *of *f*. For example, let  where(3)

Then *x*_1 _depends on both *x*_1 _and *x*_2_, while *x*_2 _depends only on *x*_1_.

While inferring the dependency graph from a PDS model is straightforward, identifying that single model may hardly be possible. There is a problem originating from the algorithm proposed in [34]: finding all polynomials that vanish on a set of points. This is equivalent to computing the ideal of these points and computation of an ideal of points boils down to intersection of ideals of polynomials vanishing on one point. There is a well-known consequence of the Buchberger algorithm, originally presented in [57] MISSING, for their computation. The output of the algorithm is a Gröbner basis {*g*_1_, ..., *g_s_*} ⊂ *k*[*x*_1_, ..., *x_n_*] that generates the ideal of vanishing polynomials: , where *h_i _*∈ *k*[*x*_1_, ..., *x_n_*]. The Gröbner basis, however, is not unique, as it was discussed in 2.1, and its computation depends on the choice of monomial ordering.

*Example ***3.1 **Consider a network of 3 genes *x*_1_, *x*_2_, and *x*_3_. Suppose we have the following time series of network states in : **s**_1 _= (2, 1, 0), **s**_2 _= (1, 2, 0), **s**_3 _= (2, 1, 1), **s**_4 _= (0, 0, 1).

Depending on the selection of monomial ordering, the algorithm of [34] will generate one of the two polynomial models:(4)

Notice that all three coordinate polynomials involve *x*_3 _but depending on the monomial ordering, they also contain either *x*_1 _or *x*_2_. In fact, for the given time series **s**_1_, ..., **s**_4_, these are the only two distinct minimal (in the sense defined in [34]) PDS models that the algorithm generates. While it is not clear whether there is a dependence on *x*_1 _or on *x*_2_, one can be confident that, provided the data are representative of the network, *x*_3 _has a definite impact on all three genes. We expand on this idea in the next section.

Clearly the monomial ordering selection affects not only the dependency graph of the model but also its dynamics which is represented by the model's state space. Let **p **= (1, 0, 0) . In Example 3.1, starting at state **p**, the first model will transition to state (0, 0, 2), while the second's next state is (2, 1, 1). All coordinates of the output are different although they are generated by minimal PDS models that fit the experimental time series data. It is not clear how to select one out of, as it is often the case with real data, hundreds of possibilities. The main reason is that it is hard to relate monomial ordering to biology in any meaningful way. The solution we propose is instead of trying to single out one model, to use all minimal models (possibly with different probabilities) to generate the system dynamics. This means allowing stochastic behavior which is consistent with the experimental observations.

**Figure 3:**
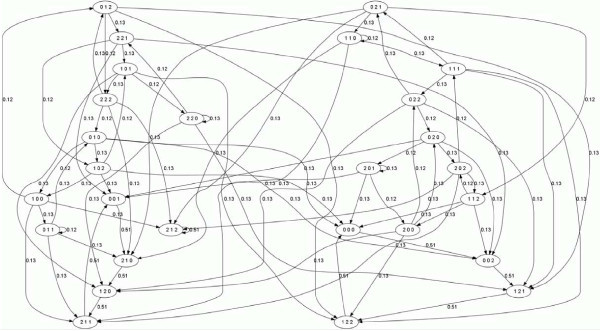
**The state spaces of PPDS (5)**.

For Example 3.1, Algorithm 3.1 generates output *F *= {*f*_1_, *f*_2_, *f*_3_} given in (5). Its state space consists of one connected component (of size 27), and five fixed points, (0, 1, 1), (1, 1, 0), (2, 0, 1), (2, 1, 2), and (2, 2, 0), with stabilities (probability that for a given run of the simulation the point will be fixed) 0.12, 0.12, 0.13, 0.51, and 0.13, respectively. Figure 3 shows the state space graph of (5).(5)

**Relative size of the Gröbner cones and interaction strength**. Using Algorithm 3.1 for reverse engineering of PPDSs is based upon the following assumptions that relate the network dependencies to the Gröbner fan of the ideal of polynomials that vanish on the network data.

1. *Minimal polynomials are an appropriate framework for the modeling of gene regulatory networks*. The minimal polynomials are only a subset of all polynomials that fit a given data set. In Example 3.1, both *f*_1 _= *x*_2 _- *x*_3 _and  fit the data and can be coordinate polynomials for *x*_1 _but the latter is not minimal since  is identically zero on the data and . In that sense, *g *is not supported by the data and is meaningless for the purpose of reverse engineering.

2. *The strength of dependency of network node x_i _on node x_j _on the scale from 0 to 1 is proportional to the relative frequency with which x_j _appears in x_i_'s minimal coordinate polynomials that fit the data*. In Example 3.1, both possible polynomials for *f*_1 _involve *x*_3 _which means that the behavior of *x*_1 _cannot be described without involving *x*_3_. It is reasonable then to conclude that the strength of the dependency of *x*_1 _on *x*_3 _is 1.

3. *The strength of dependency of x_i _on x_j _is proportional of the size of the portion of the Gröbner fan that corresponds to those coordinate polynomials for f_i _that involve x_j_*.

Each point of the Gröbner fan corresponds to a monomial ordering and all points within the same Gröbner cone produce the same Gröbner basis and thus the same minimal model. Typically, we do not know which monomial ordering(s) are more appropriate for a particular network and the best we can do is consider all of them and identify which ones are the most likely to represent the network dependencies. If there are two models, one corresponding to a larger portion of the Gröbner fan than the other, this means that there are more monomial orderings which produce the first model.

In Example 3.1, the Gröbner fan is three-dimensional since there are three variables and consists of two Gröbner cones of equal sizes (volumes). Each one of the two coordinate polynomials for *f*_1_, for instance, corresponds to .5 of the total size of the Gröbner fan. Therefore, under our assumption, the strength of dependency of *x*_1 _on *x*_2 _is .5 and the probability that *x*_1 _depends on itself is also .5.

### 4 Reverse engineering the yeast cell cycle

Based on the data generated from the model presented in [17] and given in Table 1, the reverse engineering method we proposed generated the PPDS (6). For each variable *x_i_*, the set *f_i _*consists of pairs of the form (*F*, *p*), where *F *is a polynomial in  and *p *is the probability with which *F *is used to determine *x_i_*'s next value at each iteration.(6)
